# A New Type of Chronic Wound Infection after Wisdom Tooth Extraction: A Diagnostic Approach with 16S-rRNA Gene Analysis, Next-Generation Sequencing, and Bioinformatics

**DOI:** 10.3390/pathogens9100798

**Published:** 2020-09-28

**Authors:** Sebastian Böttger, Silke Zechel-Gran, Philipp Streckbein, Michael Knitschke, Torsten Hain, Markus Weigel, Jan-Falco Wilbrand, Eugen Domann, Hans-Peter Howaldt, Sameh Attia

**Affiliations:** 1 Department of Oral and Maxillofacial Surgery, Justus-Liebig-University Giessen, University Hospital Giessen and Marburg, Location Giessen, Klinikstrasse 33, D-35392 Giessen, Germany; Philipp.Streckbein@uniklinikum-giessen.de (P.S.); Michael.Knitschke@uniklinikum-giessen.de (M.K.); hp.howaldt@uniklinikum-giessen.de (H.-P.H.); sameh.attia@dentist.med.uni-giessen.de (S.A.); 2 Institute of Medical Microbiology, Justus-Liebig-University Giessen, Biomedical Research Facility Seltersberg (BFS), Schubertstrasse 81, D-35392 Giessen, Germany; Silke.Zechel@mikrobio.med.uni-giessen.de (S.Z.-G.); Torsten.Hain@mikrobio.med.uni-giessen.de (T.H.); Markus.Weigel@mikrobio.med.uni-giessen.de (M.W.); Eugen.Domann@mikrobio.med.uni-giessen.de (E.D.); 3 German Center for Infection Research (DZIF), Partner Site Giessen-Marburg-Langen, Schubertstrasse 81, D-35392 Giessen, Germany; 4 Department for Cranio-Maxillofacial Surgery-Plastic Surgery, Diakonie Klinikum Jung-Stilling, Wichernstr. 40, D-57074 Siegen, Germany; jan-falco.wilbrand@uniklinikum-giessen.de

**Keywords:** 16S-rRNA gene analysis, third molar surgery, environmental bacteria, postoperative wound infection

## Abstract

Delayed-onset infections are rare postoperative complications of lower third molar extractions. This article presents a case of a chronic combined hard and soft tissue infection after the extraction of a third molar, where the causative organisms could only be elucidated by molecular methods. Experimental 16S-rRNA gene analysis with next-generation sequencing and bioinformatics was used to identify the bacterial spectrum of the infection. 16S-rRNA gene analysis delivered the microbiome of the abscessing inflammation while standard culture and laboratory examinations were all sterile. The microbiome showed a mixed bacterial infection with a dominance of *Delftia* and *Alcanivorax* (spp.) besides other bacteria of the normal oral flora. Using 16S-rRNA-gene analysis, next-generation sequencing, and bioinformatics, a new type of chronic wound infection after wisdom tooth extraction was found. The property of *Delftia* and *Alcanivorax* (spp.) as water-affine environmental bacteria raises suspicion of infection from contaminated water from a dental unit. Thus, osteotomies of teeth should only be done with sterile cooling water. The 16S-rRNA gene analysis should become a part of the routine diagnostics in medical microbiology.

## 1. Introduction

Bacterial infections in the field of oral and maxillofacial surgery are very common. With the application of antibiotics, the implementation of incision and drainage of abscesses, and with the removal of infected teeth, most infections can usually be treated fast and successfully [1,2,3,4,5]. Nevertheless, sometimes persistent subacute and chronic infections occur, where adequate therapy can be a great challenge for practitioners and where treatment can take a long time [6]. Especially, delayed-onset wound infections after third molar extraction, which occur several weeks after suture removal and after discharge [7], can cause varying symptoms affecting hard and soft tissues [7,8,9,10,11]. Figueiredo et al. reported that those infections are normally caused by anaerobic bacteria of the oral flora, e.g., *Fusobacterium, Peptostreptococcus*, *Porphyromonas,* and *Prevotella* strains, which in most cases can be successfully treated with combinations of surgical revision and antibiotics [9]. Other authors have shown that specific infections like Actinomycosis, which need specific therapy, have to be considered [6,12,13]. Additionally, cases of osteomyelitis of the jaw after third molar surgery have been previously described [14]. As delayed-onset infections occur only several weeks post-intervention [7,10], other diseases of non-odontogenic origin like atypical mycobacteriosis, tuberculosis, or cat scratch disease, which can present with similar symptoms, also have to be taken into account [15,16].

The present study shows a new type of delayed-onset wound infection after third molar surgery. This infection was resistant to antibiotic therapy and surgical revision. It was not possible to clarify the cause with standard culture and laboratory examinations. The study aimed to clarify this enigmatic infection by using 16S-rRNA gene analysis with next-generation sequencing and bioinformatics.

## 2. Patient and Methods

A 23-year-old woman in otherwise good general condition without any pre-existing illness visited the ambulance of oral and maxillofacial surgery at the university hospital. She suffered from inflammatory swelling and pain in the area of the wisdom tooth 38, which had been extracted by her dentist in a dental practice six months ago. After a post-operative symptom-free interval of several weeks, renewed swelling in the area of the extracted tooth and the adjacent cheek appeared. Thus, the dentist decided to start an antibiotic therapy on the patient, which was unsuccessful as an oral abscess formed, which was incised and drained. Since all attempts to treat the delayed-onset infection failed, the patient was referred to the university hospital.

**Figure 1:**
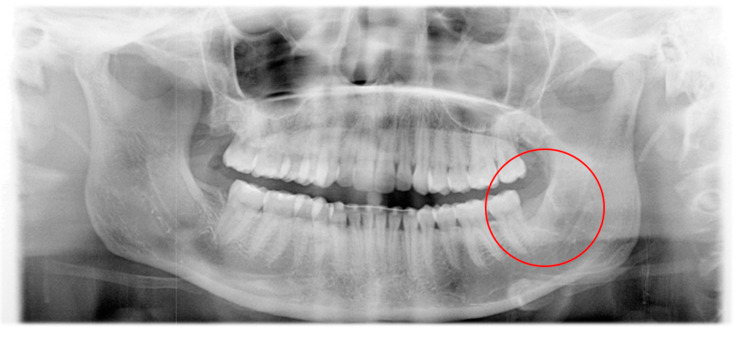
Panoramic radiograph at the first consultation. Alveolus of tooth 48 shows complete healing while ossification is missing in the area of tooth 38.

At the first consultation, chronic inflammatory and rough tissue could be investigated in the area of the former tooth 38 without evidence of abscessing. Panoramic radiograph (Figure 1) showed a non-ossified alveolus of tooth 38 while the other alveoli of the wisdom teeth were already ossified regularly.

With the suspicion of an inflammatory tumor, a surgical incision was first carried out and tissue samples were harvested for histopathologic analysis. Histopathologic investigation revealed chronic inflammation with the formation of histiocytic and epithelioid cell granuloma. Molecular testing with polymerase chain reaction (PCR), especially the IS6110 profile, was unable to identify mycobacterial DNA fragments and there was no hint for an actinomycosis.

**Figure 2:**
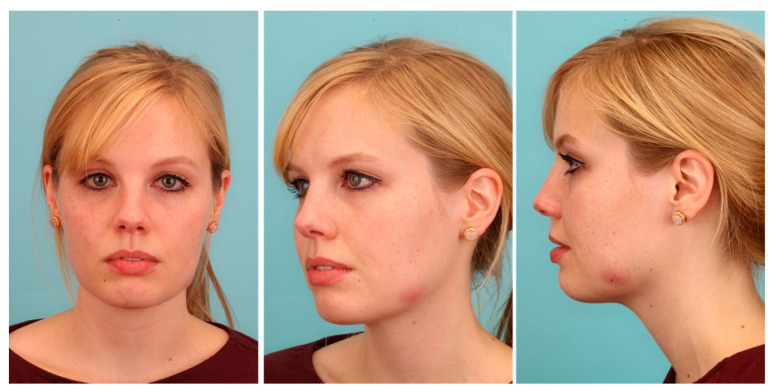
Development of an extra-oral swelling and redness despite surgical therapy.

Wound revision led to a temporary improvement of the patient’s complaints. However, after three weeks, the patient suffered from a renewed flare of the inflammation process with a rough extraoral swelling on the left cheek (Figure 2).

**Figure 3:**
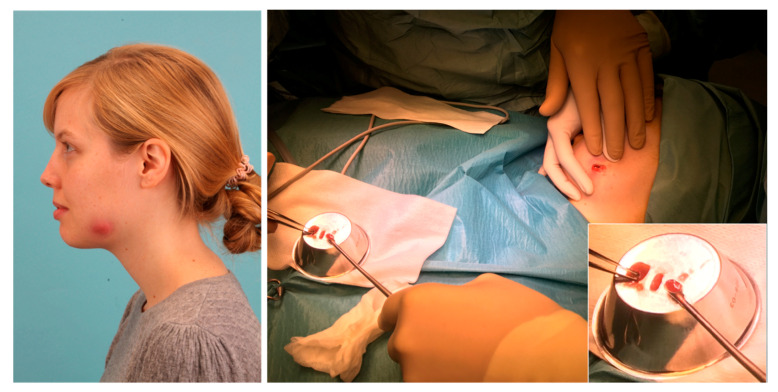
Red swelling of the left low mandible after five days of shortwave radiation therapy (**left**) and an interoperative picture of abscess incision (**right**).

Since antibiotic therapy failed, we decided to perform shortwave radiation (10 min; 5 times a day) with the intention of a purulent melting of the inflammation. After five days of therapy, heavy inflammatory signs could be observed. So, we decided to carry out an abscess incision in general anesthesia (Figure 3).

During surgery, microbiological swabs from pus and native wound tissue were taken and subjected to microbiological culture. A further tissue sample was taken for a histopathological examination and some pus was preserved in a cuvette in a medical freezer at −80 degrees centigrade (°C) for later experimental use.

Matching the clinical course, histopathological examination delivered the description of a chronically granulating partially purulent inflammation of the soft tissue, but there were no histopathological hints for actinomycosis. Molecular testing of the IS6110 profile for mycobacterial DNA fragments (*M. tuberculosis*, *M. bovis, M. bovis BCG*, *M. microti, M. africanum*) was negative again. Microbiological culture was not able to detect any aerobic or anaerobic bacteria and the culture on *Actinomycetes* was sterile too. Additionally, the culture on atypical mycobacteria was sterile after multiple weeks of culturing. Serological tests for *Bartonella henselae* and *Toxoplasma gondii* as well as a Quantiferon Gold for tuberculosis testing were also negative. Thus, the cause of the infection remained unclear despite the numerous examinations.

**Figure 4:**
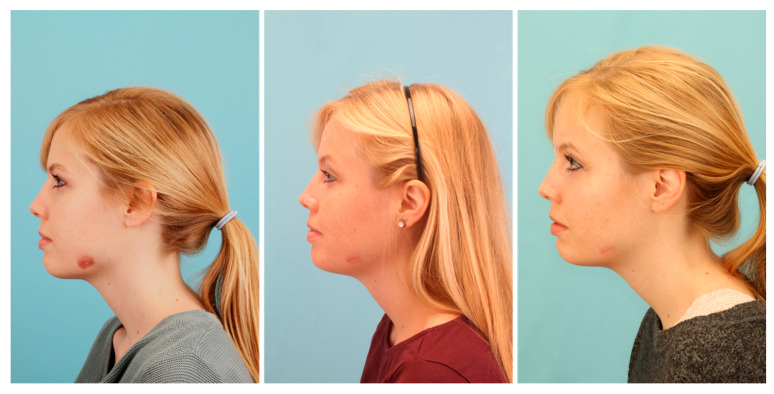
Patient profile depicting the clinical picture of the infection before long-term antibiotic therapy (**left**), after three months of long-term penicillin application (**middle**), and after the termination of therapy after six months (**right**).

The patient was released from hospital two days after surgery. Postoperatively, we applied a calculated antibiotic therapy with Sultamicillin (750 mg BID) for 10 days. Nevertheless, healing of the inflammation could not be observed in the following weeks (Figure 4). Thus, we presented the case in the local interdisciplinary infection board about two months after surgery. Since all cultures were negative and even molecular and laboratory testing was inconspicuous, we decided to treat the infection as a soft tissue actinomycosis with Penicillin V (1.5 Mega TID) for six months.

**Figure 5:**
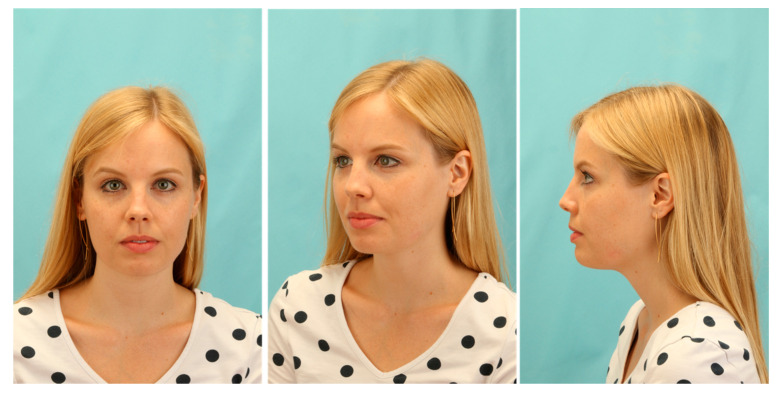
Final result: situation after 2.5 years after the first consultation.

Applying the long-term antibiotic therapy, slow recovery with fading of the inflammatory redness could be observed (Figure 4). After six months, only a small reddish scar without signs of inflammation remained (Figure 4). Thus, we decided to discontinue the antibiotic therapy and to observe the situation for the next months. We did not observe a relapse during the next six months and we were able to complete the case at about one year after the first consultation. Only a small white scar of rough consistency remained in the area of former inflammation (Figure 5). Although our therapy was successful, the triggering cause was still unknown. Only by determining the microbiome of the pus by a subsequent experimental trial, we could retrospectively clarify the cause of this extraordinary delayed-onset infection after third molar surgery.

The determination of the microbiome was carried out with 16S-rRNA amplicon sequencing and bioinformatics [17]. In prokaryotes, the 16S-rRNA gene carries the genetic information of the 16S ribosomal ribonucleic acid of the 30S subunit of the ribosome. This gene consists of variable and conserved areas. Conserved areas are not different between various bacterial genera while variable areas have high specificity for different bacterial genera. Based on the sequence of the variable areas of the gene, it is possible to assign bacterial DNA to a specific genus. Thus, 16S-rRNA amplicon sequencing works like a fingerprint, if the base sequence can be matched with a ribosomal database. It is particularly useful in identifying unusual bacteria that are difficult to identify by conventional methods, providing genus identification in >90% of cases [18]. In our case, bacterial DNA was first extracted from the initially frozen pus. Then, the variable area “V4” of the 16S-rRNA-genes was amplified by PCR using primers in the conserved flanking areas. The resulting amplicons of an approximate length of 350 to 370 bps were then equipped with indices and adapters for next-generation sequencing using the Illumina MiSeq System as described by the vendor (Illumina). After the sequencing of these amplicons, the bacteria-specific sequences were known and ready for bioinformatic analysis.

Bioinformatic analysis was carried out with the open-source software “Mothur” (mothur.org) and the SILVA ribosomal RNA gene database (www.arb-silva.de). In this way, the assignment of the amplicon sequence to the respective bacterial gene was managed, which enabled the determination of the microbiome of the pus sample. In addition to this qualitative identification, the frequencies of the identified amplicons were detected. With this quantitative approach, it was possible to conclude the frequencies of the individual bacterial genera in the pus sample.

After sequencing on the MiSeq platform using the MiSeq Reagent Kit v2, a total of 314,578 paired end reads with a length of about 250 nucleotides (nt) were obtained. As mentioned above, microbiome analysis was executed using Mothur (https://mothur.org/wiki/miseq_sop/, accessed on 20/09/2017) [19]. Paired end reads were joined, primer regions removed, and filtered for the expected amplicon length of 253 nt ± 10 nt excluding sequences that contained ambiguous nucleotides. Joined paired end reads were aligned to the SILVA ribosomal RNA gene database [20], trimmed to contain only the hypervariable regions V4, and clustered with a similarity threshold of 97%. After chimera removal using the Mothur implementation of VSEARCH [21], a total of 59 operational taxonomic units (OTUs) representing 681 paired end reads were obtained. OTUs were finally classified against the SILVA ribosomal RNA gene database and exported in csv format.

Tables and graphs were created with Microsoft Excel 2016 and Microsoft Office Professional Plus 2016 (Microsoft, Washington, WA, USA). 

The investigations used in this study were approved by the ethics committee of the Faculty of Medicine at Justus-Liebig University (Giessen, Germany; approval no. 191/16).

## 3. Results

**Figure 6:**
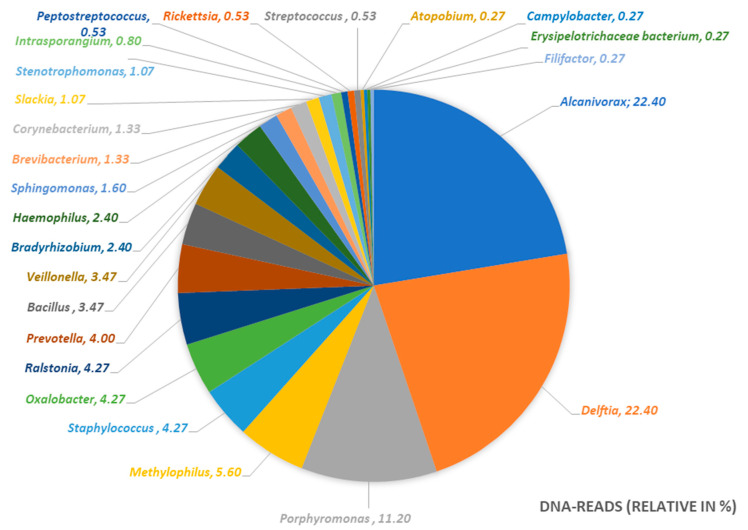
The microbiome of the pus sample. Relative frequencies show a predominance of *Alcanivorax*, *Delftia*, and *Porphyromonas* (spp.).

**Figure 7:**
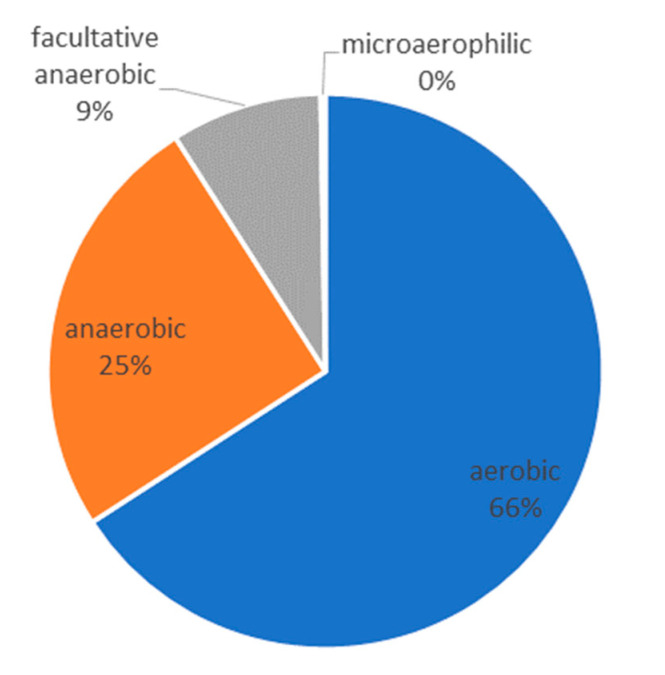
Metabolism of the bacterial genera in the pus sample. The identified bacteria predominantly have aerobic metabolism.

The result of the 16S-rRNA gene sequencing elucidated the microbiome of the abscess. Although cultures were sterile, the microbiome shows a dominance of the genera *Delftia*, *Alcanivorax*, and *Porphyromonas*. An infection with *Actinomycetes* or atypical Mycobacteria could be excluded. We could identify and prove a polymicrobial infection with a dominance of aerobic water-affine environmental bacteria as the cause of the delayed-onset infection after third molar surgery in the here discussed case (Table 1). The results are presented in a pie chart with relative frequencies (Figure 6). Figure 7 shows the metabolism of the bacterial genera in the pus sample.

## 4. Discussion

Wound infections after third molar surgery can be classified into early onset infections and delayed-onset infections. While early onset infections occur in the early healing phase, delayed-onset infections do not appear until a time of about four to six weeks post-surgery and are almost only seen in the lower jaw [11]. The probability of the occurrence of a delayed-onset infection after third molar surgery is calculated with a frequency of 0.49% to 2.2% [7,11]. The cause of such infections often cannot be clarified [7,11]. Possible causes of infection include food impaction into the wound [11] and the formation of a hematoma under the flap [22]. Risk factors described in the literature include the depth of inclusion of wisdom teeth [7,22,23], lack of distal space [8], a vertical or mesioangular tilt of the tooth [8], the need for intraoperative hemostasis [11], and total and tight wound closure [9]. Additionally, the changes in oral flora caused by systemic and topical antibacterial therapy could favor the development of opportunistic infections [7,24], which can result in a higher postoperative infection rate [25]. It was pointed out that in the context of abscess formation, improper use of antibiotics can even increase the risk of a chronification of inflammation [4]. 

As causal pathogens, Figueiredo et al. most frequently identified *Fusobacterium* sp., *Prevotella* sp., and *Peptostreptococcus* sp. in delayed-onset infections after lower third molar surgery [9]. Thus, typical anaerobic bacteria of the oral cavity seem to play a major role in delayed-onset infections [9]. The fact that for all patients of this study multiple bacteria could be found shows that delayed-onset infections are polymicrobial infections, in which rare bacteria like *Actinomyces israelii* can also be found [9]. In another work, Figuereido et al. described that about two-thirds of the patients with delayed-onset infections could be successfully treated with antibiotics alone [8]. One third, however, needed an additional surgical procedure in terms of a wound revision and this was particularly true after a prolonged period of initial postoperative antibiotic treatment [8]. The fact that also persistent chronic infections with a very difficult treatment were reported [6,14,26,27] points out that in such cases, the practitioner is occasionally confronted with a difficult diagnostic and therapeutic situation.

The here presented patient developed a delayed-onset infection in the left third molar region some weeks after an initially unremarkable course. Since antibiotic therapy and surgical revision failed, we decided to discontinue antibiotic treatment and to start shortwave radiation with the goal of a purulent melting of the process instead. After five days, an abscessing formation was seen on clinical examination, which allowed incision and acquisition of pus samples, smears, and tissue samples for microbiological and pathological examination. As described above, cultures for aerobe and anaerobe bacteria were negative and even special cultures for *Actinomyces* and atypical mycobacteria did not reveal any bacterial growth. Numerous authors have pointed out that diagnosing actinomycosis can be a great challenge [6,12,13,28]. Successful isolation and identification of these bacteria only occurs in a minority of cases. The rate of falsely negative cultures is high due to previous antibiotic therapy, inhibition of *Actinomyces* growth by concomitant microorganisms or contamination, inadequate culture conditions, or inadequate short-term incubation [12,28]. Due to the long atypical course of the chronic disease, infection with atypical mycobacteria was also considered in the differential diagnosis [15]. These infections present with non-tender unilateral lymphadenopathy in otherwise healthy patients. Initially, this disease may be mistaken for a staphylococcal or streptococcal infection leading to inappropriate incision and drainage, which can cause cosmetic complications as in the present case [16]. 

In the here described case, neither microbiological nor histopathological nor serological examinations led to a diagnosis of a specific infection. Thus, antibiotic therapy was finally carried out in a calculated manner, which resulted in a cure after several months. However, the correct diagnosis could only be found afterwards using an experimental 16S-rRNA gene analysis with next-generation sequencing and bioinformatics as an additional tool. In this way, the microbiome of the pus sample from the abscessing inflammation was determined. As shown in Table 1 and Figure 6, the disease of the present case was not caused by one specific bacterium. As described in the work of Figueiredo et al., we found a polymicrobial infection as the reason for the delayed-onset infection after third molar surgery [9]. Apart from the typical bacterial spectrum of odontogenic infections like *Peptostreptococcus* (spp.), *Porphyromonas* (spp.), or *Prevotella* (spp.), we observed that almost 56% of the DNA originated from the genera *Alcanivorax*, *Delftia* [9,29,30], and *Porphyromonas*. According to our research, no human pathogenicity has yet been demonstrated for *Alcanivorax* while numerous diseases have been linked to *Delftia*, especially to *Delftia acidovorans*. Even if no cultural evidence for such a polymicrobial infection can be provided and the abundance of bacteria in the microbiome of the pus alone does not provide any evidence of pathogenicity of individual bacteria, the findings of the here described case suggest that the patient did not have a typical odontogenic infection. The following three points should indicate that we have found a separate disease entity:

1. The infection of the patient was completely atypical from the clinical point of view. A typical odontogenic infection can usually be managed well with surgery and antibiotic therapy. In the present case, it took more than a year to cure the patient.
2. In the case of a normal odontogenic infection, bacteria can usually be cultured from the pus. However, the culture, in this case, was negative, which is probably because the cultural conditions were not optimal or sufficient for the later detected atypical bacterial genera.
3. The determined microbiome was completely different from the one we would expect in a normal odontogenic abscess. As described by Figueiredo et al. [9], odontogenic infections show a polymicrobial spectrum with mostly obligate anaerobic bacteria. In the microbiome of the abscess of the present case, we mostly found aerobic environmental bacteria in combination with anaerobic bacteria of the oral cavity. Taking into account that the pus sample was taken from an extra-oral incision, we assume that the infection can be caused by a combination of these bacteria.

*Delftia acidovorans* is an aerobic, non-fermentative, Gram-negative rod that is classified in the *Pseudomonas* rRNA homology group III [31]. It is usually a non-pathogenic environmental organism that rarely is clinically significant [32]. Although *Delftia acidovorans* infections most commonly occur in hospitalized or immunocompromised patients, there are several reports documenting the infection in immunocompetent patients [32]. In this context, diseases like pneumonia, intravascular catheter-associated bacteremia, endocarditis, peritonitis, ocular infections, and urinary tract infections have been reported in the literature [31,32,33,34,35,36]. Due to its attribute as an environmental organism with a great affinity to water and damp surroundings [37], its occurrence was also described for water distribution systems in medical institutions [38]. It was also proven that *Delftia acidovorans* and other species of the *Pseudomonadaceae* family are often found in biofilms of dental unit waterlines and that they are prone to be transmitted with aerosols and splatters, generated by working unit handpieces [39].

The species of *Alcanivorax* was first described by Yakimov et al., who described *Alcanivorax borkumensis* as a hydrocarbon-degrading and surfactant-producing marine environmental bacterium [40]. It became known as an oil-degrading bacterium in oil spills [41,42,43].

With *Delftia* and *Alcanivorax* (spp.), two water-affine environmental bacteria were identified as dominant rods in the pus sample of the delayed-onset infection after third molar surgery in the present case. It could be argued that this was a result of the contamination of the pus sample. However, since the sample was taken under strictly aseptic conditions in the operating room and without the use of cooling water (Figure 3), we suspect that contamination with environmental bacteria is very unlikely. Numerous authors have reported that *Delftia* (spp.) can be found in dental unit waterlines [39,44]. With the lubricant oil of working unit handpieces, the main nutrient for *Alcanivorax* was also present. It can be suspected that these environmental bacteria were transferred during the initial removal of the wisdom tooth or in the context of a surgical revision and that these bacteria developed a symbiosis with other bacteria of the microbiome of the oral cavity. To our knowledge, this is the first case of such an odontogenic polymicrobial infection with a dominance of the environmental bacteria *Delftia* (spp.) and *Alcanivorax* (spp.). Since no human pathogenicity has yet been reported for *Alcanivorax* (spp.) and as *Delftia* (spp.) has often been associated with infections in immunosuppressed individuals, we want to emphasize that in the present case the infection was observed in a young and otherwise healthy woman without any pre-existing diseases [32,34,37]. It cannot be excluded that there is a real risk for an opportunistic bacterial infection from dental unit waterlines even in young and healthy patients if possibly harmless environmental bacteria are transferred to an open wound. This results in the recommendation to perform invasive procedures, like the osteotomy of an impacted wisdom tooth, only with sterile cooling water and not with the water of a normal dental unit. Even if the risk of infection from a dental unit seems to be very low, the present case shows that in some cases perioperative wound infections with normally harmless environmental bacteria are possible, even in immunocompetent patients. If such a wound infection occurs, it can be difficult to find the correct diagnosis and to find a suitable therapy. In the present case, it finally took six months to heal the patient.

In the future, 16S-rRNA gene analysis could be integrated into the normal clinical routine. This could facilitate the diagnosis of atypical odontogenic infections when the disease-causing bacteria are difficult to cultivate. Furthermore, 16S-rRNA gene analysis seems to provide a more comprehensive and complete picture of the involved bacteria than previously possible with the use of cultural methods only. Further studies on the analysis of odontogenic abscess microbiomes could increase our knowledge and facilitate diagnosis and advance treatment options.

## 5. Conclusions

The cause of a persistent delayed-onset infection after third molar surgery could in the present case only be clarified by experimental use of 16S-rRNA gene analysis with next-generation sequencing and bioinformatics. A new type of chronic wound infection with a predominance of the environmental bacteria *Delftia* and *Alcanivorax* (spp.) in combination with an odontogenic polymicrobial flora was found. Since there is reasonable suspicion that the environmental bacteria were transferred from a dental unit waterline, we recommend to always perform invasive oral surgical procedures with sterile cooling water. The 16S-rRNA gene analysis should become a part of the routine diagnostics in medical microbiology.

## Figures and Tables

**Table 1:** Relative frequency of DNA reads in the pus sample.

Bacterial Genus	DNA-Reads (Relative in %)	Metabolism
Alcanivorax	22.40	aerobic
Delftia	22.40	aerobic
Porphyromonas	11.20	anaerobic
Methylophilus	5.60	aerobic
Staphylococcus	4.27	facultative anaerobic
Oxalobacter	4.27	anaerobic
Ralstonia	4.27	aerobic
Prevotella	4.00	anaerobic
Bacillus	3.47	aerobic
Veillonella	3.47	anaerobic
Bradyrhizobium	2.40	aerobic
Haemophilus	2.40	facultative anaerobic
Sphingomonas	1.60	aerobic
Brevibacterium	1.33	aerobic
Corynebacterium	1.33	facultative anaerobic
Slackia	1.07	anaerobic
Stenotrophomonas	1.07	aerobic
Intrasporangium	0.80	aerobic
Peptostreptococcus	0.53	anaerobic
Rickettsia	0.53	aerobic
Streptococcus	0.53	facultative anaerobic
Atopobium	0.27	facultative anaerobic

## References

[1] Wiese K.G., Merten H.A., Wiltfang J., Luhr H.G. (1999). Clinical studies on the pathophysiology of odontogenic abscesses. Mund Kiefer Gesichtschir..

[2] Warnke P.H., Becker S.T., Springer I.N., Haerle F., Ullmann U., Russo P.A., Wiltfang J., Fickenscher H., Schubert S. (2008). Penicillin compared with other advanced broad spectrum antibiotics regarding antibacterial activity against oral pathogens isolated from odontogenic abscesses. J. Craniomaxillofac. Surg..

[3] Al-Nawas B., Maeurer M. (2008). Severe versus local odontogenic bacterial infections: Comparison of microbial isolates. Eur. Surg. Res..

[4] Al-Nawas B., Karbach J. (2016). S3-Leitlinie (Langversion): Odontogene Infektionen. Leitlinien Zahnmedizin.

[5] Böttger S., Lautenbacher K., Domann E., Howaldt H.-P., Attia S., Streckbein P., Wilbrand J.-F. (2020). Indication for an additional postoperative antibiotic treatment after surgical incision of serious odontogenic abscesses. J. Cranio-Maxillofac. Surg..

[6] Figueiredo L.M., Trindade S.C., Sarmento V.A., de Oliveira T.F., Muniz W.R., Valente R.O. (2013). Actinomycotic osteomyelitis of the mandible: An unusual case. Oral Maxillofac. Surg..

[7] Figueiredo R., Valmaseda-Castellon E., Berini-Aytes L., Gay-Escoda C. (2005). Incidence and clinical features of delayed-onset infections after extraction of lower third molars. Oral Surg. Oral Med. Oral Pathol. Oral Radiol. Endod..

[8] Figueiredo R., Valmaseda-Castellon E., Laskin D.M., Berini-Aytes L., Gay-Escoda C. (2008). Treatment of delayed-onset infections after impacted lower third molar extraction. J. Oral Maxillofac Surg..

[9] Figueiredo R., Valmaseda-Castellon E., Formoso-Senande M.F., Berini-Aytes L., Gay-Escoda C. (2012). Delayed-onset infections after impacted lower third molar extraction: Involved bacteria and sensitivity profiles to commonly used antibiotics. Oral Surg. Oral Med. Oral Pathol. Oral Radiol..

[10] Brunello G., De Biagi M., Crepaldi G., Rodrigues F.I., Sivolella S. (2017). An Observational Cohort Study on Delayed-Onset Infections after Mandibular Third-Molar Extractions. Int. J. Dent..

[11] Sukegawa S., Yokota K., Kanno T., Manabe Y., Sukegawa-Takahashi Y., Masui M., Furuki Y. (2019). What are the risk factors for postoperative infections of third molar extraction surgery: A retrospective clinical study?. Med. Oral Patol Oral Cir. Bucal..

[12] Valour F., Sénéchal A., Dupieux C., Karsenty J., Lustig S., Breton P., Gleizal A., Boussel L., Laurent F., Braun E. (2014). Actinomycosis: Etiology, clinical features, diagnosis, treatment, and management. Infect. Drug Resist..

[13] Wong V.K., Turmezei T.D., Weston V.C. (2011). Actinomycosis. BMJ.

[14] Gonzalez-Navarro B., Arranz-Obispo C., Albuquerque R., Jane-Salas E., Lopez-Lopez J. (2017). Osteomyelitis of the jaw (with pathological fracture) following extraction of an impacted wisdom tooth. A case report. J. Stomatol Oral Maxillofac Surg..

[15] Bayazit Y.A., Bayazit N., Namiduru M. (2004). Mycobacterial cervical lymphadenitis. ORL J. Otorhinolaryngol Relat Spec..

[16] Thavagnanam S., McLoughlin L.M., Hill C., Jackson P.T. (2006). Atypical Mycobacterial infections in children: The case for early diagnosis. Ulster Med. J..

[17] Keller P.M., Hombach M., Bloemberg G.V. (2010). 16S-rRNA-Gen-basierte Identifikation bakterieller Infektionen. Biospektrum: Das Magazin für Biowissenschaften.

[18] Woo P.C., Lau S.K., Teng J.L., Tse H., Yuen K.Y. (2008). Then and now: Use of 16S rDNA gene sequencing for bacterial identification and discovery of novel bacteria in clinical microbiology laboratories. Clin. Microbiol. Infect..

[19] Kozich J.J., Westcott S.L., Baxter N.T., Highlander S.K., Schloss P.D. (2013). Development of a dual-index sequencing strategy and curation pipeline for analyzing amplicon sequence data on the MiSeq Illumina sequencing platform. Appl. Environ. Microbiol..

[20] Quast C., Pruesse E., Yilmaz P., Gerken J., Schweer T., Yarza P., Peplies J., Glöckner F.O. (2013). The SILVA ribosomal RNA gene database project: Improved data processing and web-based tools. Nucleic Acids Res..

[21] Rognes T., Flouri T., Nichols B., Quince C., Mahé F. (2016). VSEARCH: A versatile open source tool for metagenomics. PeerJ..

[22] Goldberg M.H., Nemarich A.N., Marco W.P. (1985). Complications after mandibular third molar surgery: A statistical analysis of 500 consecutive procedures in private practice. J. Am. Dent. Assoc..

[23] Christiaens I., Reychler H. (2002). [Complications after third molar extractions: Retrospective analysis of 1,213 teeth]. Rev. Stomatol. Chir. Maxillofac..

[24] Goldberg M.H., Galbraith D.A. (1984). Late onset of mandibular and lingual dysesthesia secondary to postextraction infection. Oral Surg. Oral Med. Oral Pathol..

[25] Piecuch J.F., Arzadon J., Lieblich S.E. (1995). Prophylactic antibiotics for third molar surgery: A supportive opinion. J. Oral Maxillofac Surg..

[26] Boon L.C. (1987). Late presentation of actinomycosis after third molar surgery. Med. J. Malaysia.

[27] Bonnefond S., Catroux M., Melenotte C., Karkowski L., Rolland L., Trouillier S., Raffray L. (2016). Clinical features of actinomycosis: A retrospective, multicenter study of 28 cases of miscellaneous presentations. Medicine (Baltimore).

[28] Bennhoff D.F. (1984). Actinomycosis: Diagnostic and therapeutic considerations and a review of 32 cases. Laryngoscope.

[29] Eckert A.W., Hohne C., Schubert J. (2000). Pathogen spectrum and resistance status of exclusively anaerobic odontogenic infections. Mund Kiefer Gesichtschir..

[30] Riggio M.P., Aga H., Murray C.A., Jackson M.S., Lennon A., Hammersley N., Bagg J. (2007). Identification of bacteria associated with spreading odontogenic infections by 16S rRNA gene sequencing. Oral Surg. Oral Med. Oral Pathol. Oral Radiol. Endod..

[31] Khan S., Sistla S., Dhodapkar R., Parija S.C. (2012). Fatal Delftia acidovorans infection in an immunocompetent patient with empyema. Asian Pac. J. Trop Biomed..

[32] Bilgin H., Sarmis A., Tigen E., Soyletir G., Mulazimoglu L. (2015). Delftia acidovorans: A rare pathogen in immunocompetent and immunocompromised patients. Can. J. Infect. Dis Med. Microbiol..

[33] Kawamura I., Yagi T., Hatakeyama K., Ohkura T., Ohkusu K., Takahashi Y., Kojima S., Hasegawa Y. (2011). Recurrent vascular catheter-related bacteremia caused by Delftia acidovorans with different antimicrobial susceptibility profiles. J. Infect. Chemother..

[34] Chotikanatis K., Backer M., Rosas-Garcia G., Hammerschlag M.R. (2011). Recurrent intravascular-catheter-related bacteremia caused by Delftia acidovorans in a hemodialysis patient. J. Clin. Microbiol..

[35] Mahmood S., Taylor K.E., Overman T.L., McCormick M.I. (2012). Acute infective endocarditis caused by Delftia acidovorans, a rare pathogen complicating intravenous drug use. J. Clin. Microbiol..

[36] Lee S.M., Kim M.K., Lee J.L., Wee W.R., Lee J.H. (2008). Experience of Comamonas acidovorans keratitis with delayed onset and treatment response in immunocompromised cornea. Korean J. Ophthalmol..

[37] Camargo C.H., Ferreira A.M., Javaroni E., Reis B.A., Bueno M.F., Francisco G.R., Gallo J.F., Garcia Dde O. (2014). Microbiological characterization of Delftia acidovorans clinical isolates from patients in an intensive care unit in Brazil. Diagn. Microbiol. Infect. Dis..

[38] Muchesa P., Leifels M., Jurzik L., Hoorzook K.B., Barnard T.G., Bartie C. (2017). Coexistence of free-living amoebae and bacteria in selected South African hospital water distribution systems. Parasitol. Res..

[39] Szymanska J., Sitkowska J., Dutkiewicz J. (2008). Microbial contamination of dental unit waterlines. Ann. Agric. Environ. Med..

[40] Yakimov M.M., Golyshin P.N., Lang S., Moore E.R., Abraham W.R., Lunsdorf H., Timmis K.N. (1998). *Alcanivorax borkumensis* gen. nov., sp. nov., a new, hydrocarbon-degrading and surfactant-producing marine bacterium. Int. J. Syst. Bacteriol..

[41] Naether D.J., Slawtschew S., Stasik S., Engel M., Olzog M., Wick L.Y., Timmis K.N., Heipieper H.J. (2013). Adaptation of the hydrocarbonoclastic bacterium Alcanivorax borkumensis SK2 to alkanes and toxic organic compounds: A physiological and transcriptomic approach. Appl. Environ. Microbiol..

[42] Kasai Y., Kishira H., Syutsubo K., Harayama S. (2001). Molecular detection of marine bacterial populations on beaches contaminated by the Nakhodka tanker oil-spill accident. Environ. Microbiol..

[43] Godfrin M.P., Sihlabela M., Bose A., Tripathi A. (2018). Behavior of Marine Bacteria in Clean Environment and Oil Spill Conditions. Langmuir.

[44] Williams J.F., Johnston A.M., Johnson B., Huntington M.K., Mackenzie C.D. (1993). Microbial contamination of dental unit waterlines: Prevalence, intensity and microbiological characteristics. J. Am. Dent. Assoc..

